# Supplementary material for: Experimental and Theoretical Studies of the Optical Properties of the Schiff Bases and Their Materials Obtained from o-Phenylenediamine
Source: Molecules. 2022 Oct 31;27(21):7396. doi: 10.3390/molecules27217396 (PMC9658335; doi:10.3390/molecules27217396)
Supplement: Supplementary file 1 [file molecules-27-07396-s001.zip › molecules-1955697-supplementary.pdf]

Supplementary Materials

Experimental and Theoretical Studies of the Optical Properties of the Schiff Bases and Their Materials Obtained from o-Phenylenediamine

Magdalena Barwiolek [1,*], Dominika Jankowska [1,*] Anna Kaczmarek-Kędziera [1], Slawomir Wojtulewski [2], Lukasz Skowroński [3], Tomasz Rerek [3], Paweł Popielarski [4] and Tadeusz M. Muziol [1]

1 Faculty of Chemistry, Nicolaus Copernicus University in Torun, Gagarina 7, 87-100 Torun, Poland

2 Faculty of Chemistry, University of Białystok, Ciolkowskiego 1K, Białystok, 15-245, Poland

3 Faculty of Chemical Technology and Engineering, Bydgoszcz University of Science and Technology, Kaliskiego 7, Poland, 85-796 Bydgoszcz, Poland

4 Institute of Physics, Kazimierz Wielki University, Chodkiewicza 30, 85-064 Bydgoszcz, Poland

* Correspondence: mbarwiolek@umk.pl; Tel.: +48-56-611-4516;

Table of contents:

Figure S1. ^{1}H NMR spectrum of **L1** (500 MHZ, $CDCl_3$-d_3).

Figure S2. ^{13}C NMR spectrum of **L1** (500 MHZ, $CDCl_3$-d_3).

Figure S3. ^{1}H NMR spectrum of **L2** (700 MHZ, $CDCl_3$-d_3).

Figure S4. ^{13}C NMR spectrum of **L2** (700 MHZ, $CDCl_3$-d_3).

Figure S5. IR spectrum of **L1**, KBr.

Figure S6. IR spectrum of **L2**, KBr.

Figure S7. Structure of **L1** with two crystallographically independent molecules resulting from two halves of the macrocyclic compound found in the asymmetric unit. There is given a numbering scheme and thermal ellipsoids at 30% probability

Figure S8. Hirshfeld surfaces and fingerprints of selected interactions created in the crystal network of **L1**: a. Hirshfeld surface for H…H (red markers correspond to the shortest H…H interactions at 1.15, 1.1), b. fingerprint for H…H (51.1%), c. Hirshfeld surface for C…H, d. fingerprint for C…H (19.3%), e. Hirshfeld surface for H…C (red markers correspond mainly to the spike at 0.95; 1.5), f. fingerprint for H…C (15.2%) for

O34 molecule. In brackets, there is given surface area included as a percentage of the total surface area.

Figure S9. Structure of **L2** with a numbering scheme and thermal ellipsoids at 30% probability.

Figure S10. Hirshfeld surfaces and fingerprints of selected interactions created in the crystal network of **L2**: a. Hirshfeld surface for H…H (red markers correspond to the large spike at 1.1; 1.15), b. fingerprint for H…H (65.4%), c. Hirshfeld surface for C…H, d. fingerprint for C…H (12.4%), e. Hirshfeld surface for H…C, f. fingerprint for H…C (10.5%) for O34 molecule. In brackets, there is given surface area included as a percentage of the total surface area.

Figure S11. Frontier molecular orbitals of **L1** for the most intensive transitions (PBE0/6-311++G(d,p)/PCM(acetonitrile))

Figure S12. Absorption spectrum of **L1**, **L2** calculated with PBE0/6-311++G(d,p) approach in vacuum and in solvents.

Figure S13. Frontier molecular orbitals of **L2** for the most intensive transitions (PBE0/6-311++G(d,p)/PCM(acetonitrile)).

Figure S14. SEM images of **L1**/Si maping scanning size 100 μm.

Figure S15. a), b) and c) DRIFT spectrum of **L1** and **L2** samples and **L1**/Si and **L2**/Si.

Table S1. Crystal data and structure refinement for **L1** and **L2**.

Table S2. Selected bond length [Å] and valence angles [°] for the **L1**.

Table S3. Relevant photophysical data of studied compounds, (λ_{em}, λ_{ex} nm, λ[nm] (ε [dm^3 mol^{-1} cm^{-1}]), bp=8

Table S4. Theoretical PBE0/6-311++G(d,p)/PCM(ACN) vertical excitation wavelengths λ [nm] for most intensive transitions together with the corresponding oscillator strengths f and the orbital contributions for investigated species.

Table S5. Relevant fluorescent data of studied compounds in the solid state (λ_{em}, λ_{ex}) [nm].

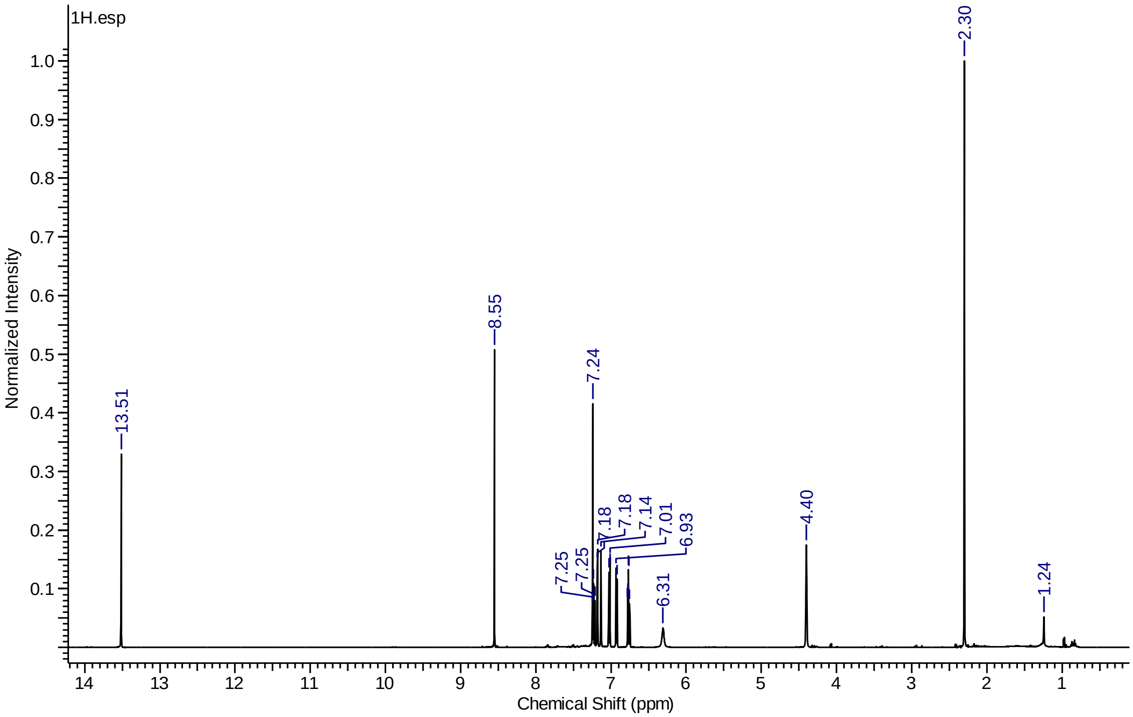

Figure S1. ^{1}H NMR spectrum of **L1** (500 MHZ, $CDCl_3$-d_3).

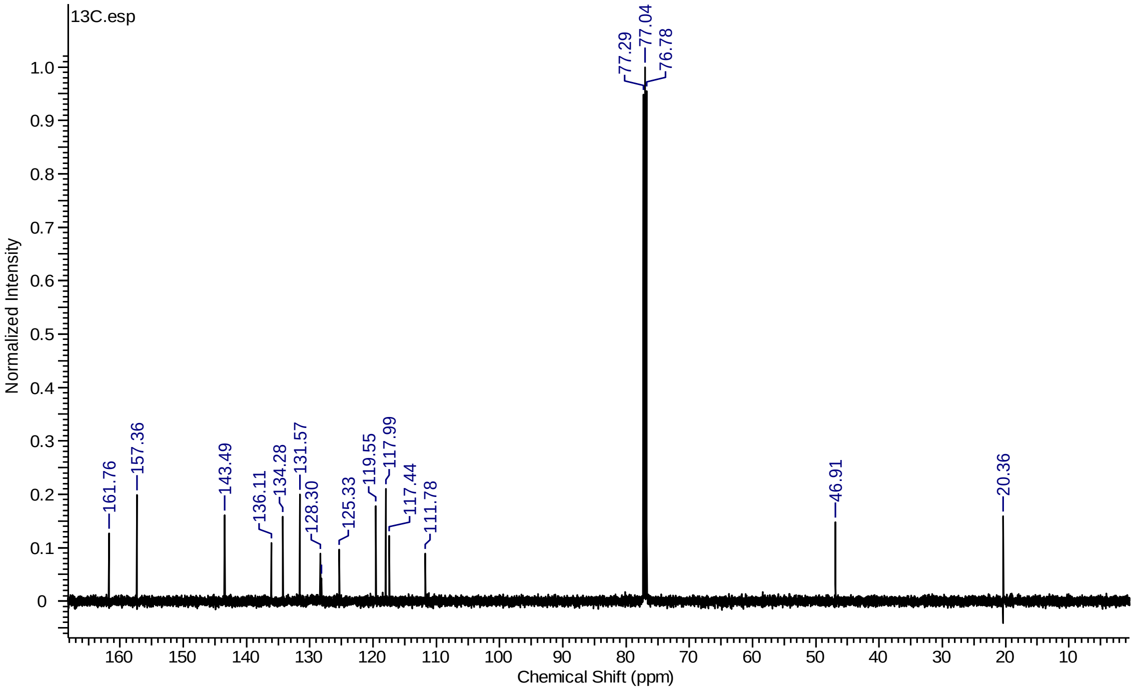

Figure S2. ^{13}C NMR spectrum of **L1** (500 MHZ, $CDCl_3$-d_3).

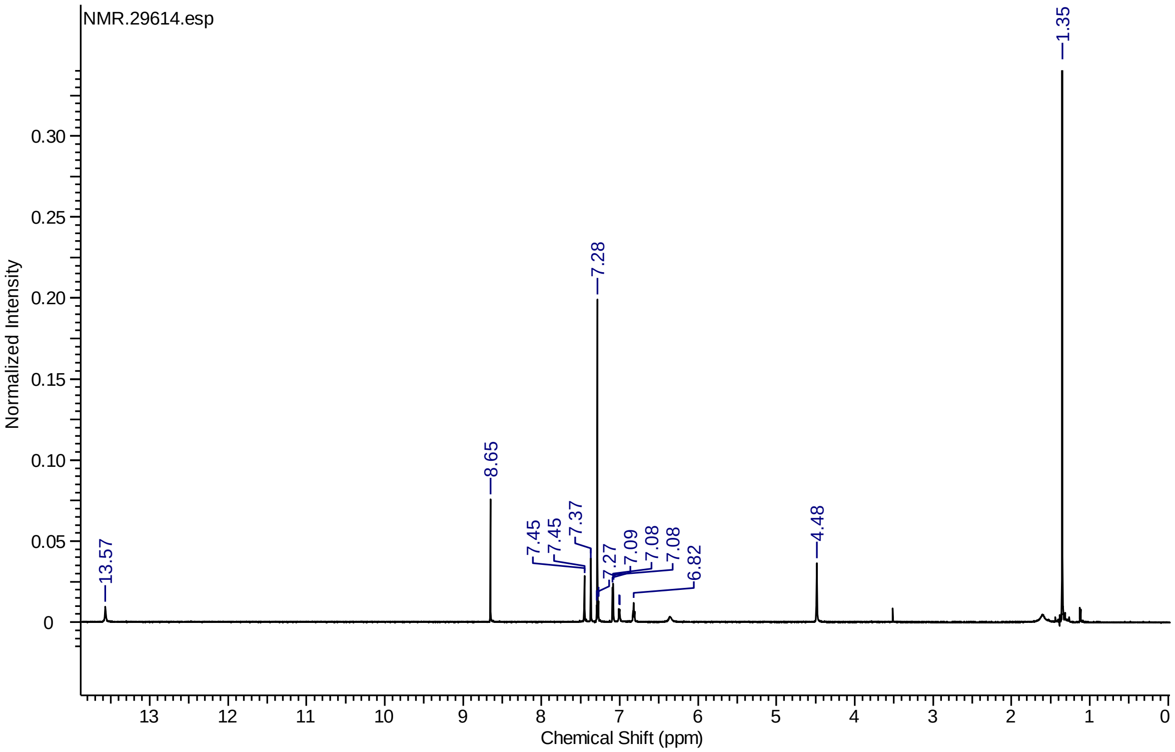

Figure S3. ^{1}H NMR spectrum of **L2** (700 MHZ, $CDCl_3$-d_3).

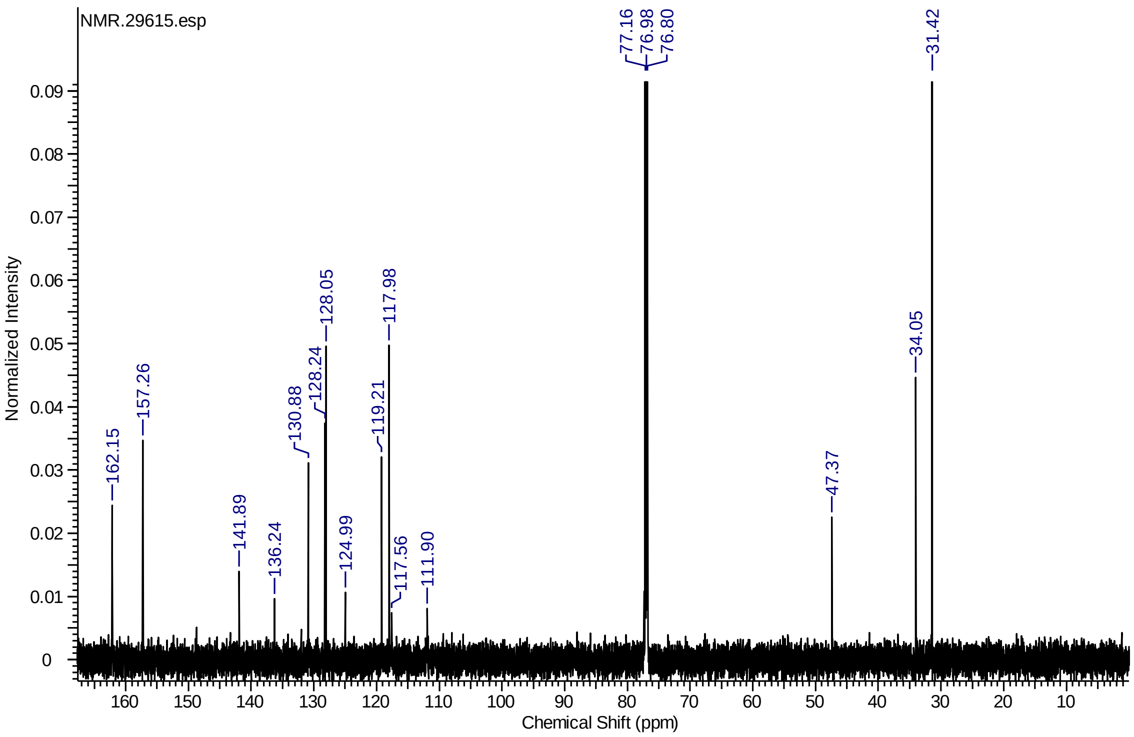

Figure S4. ^{13}C NMR spectrum of **L2** (700 MHZ, $CDCl_3$-d_3).

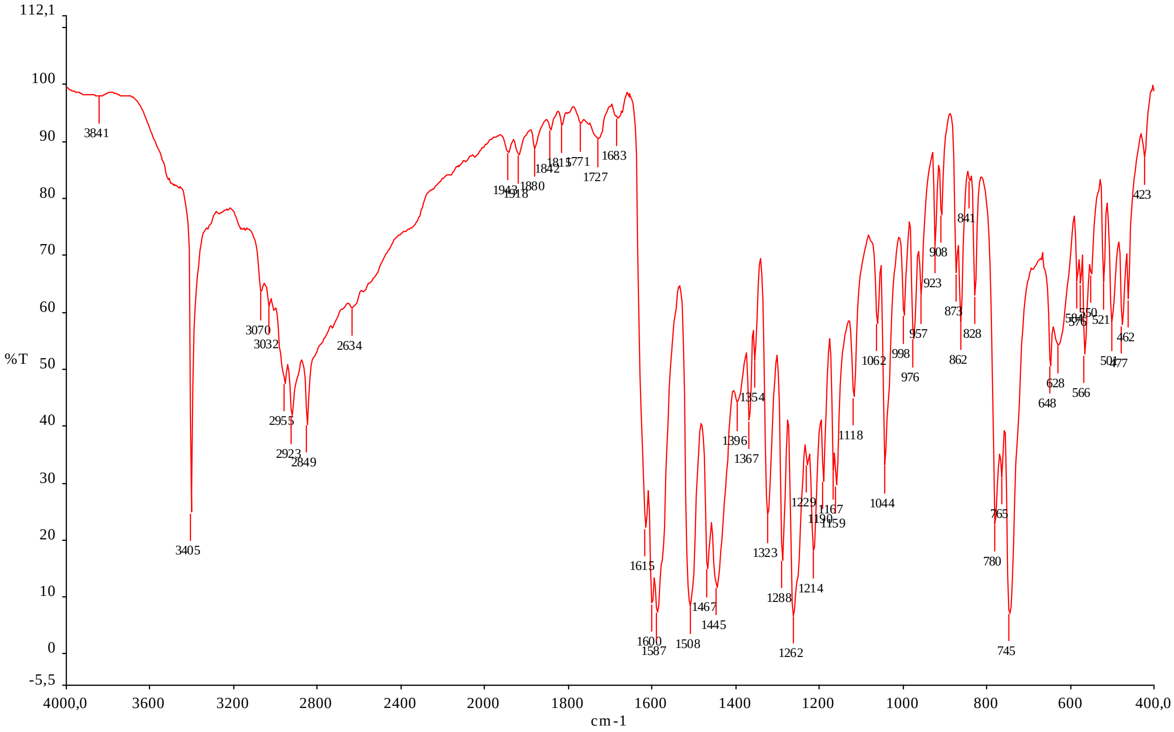

Figure S5. IR spectrum of **L1**, KBr.

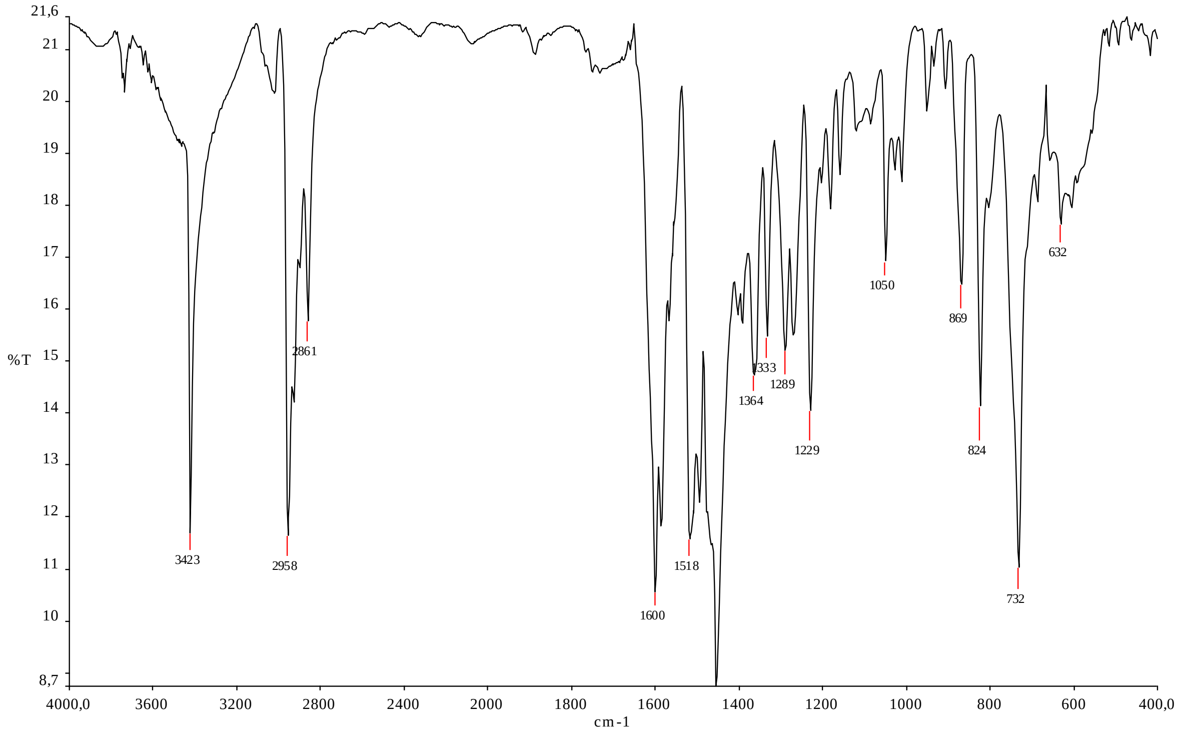

Figure S6. IR spectrum of **L2**, KBr.

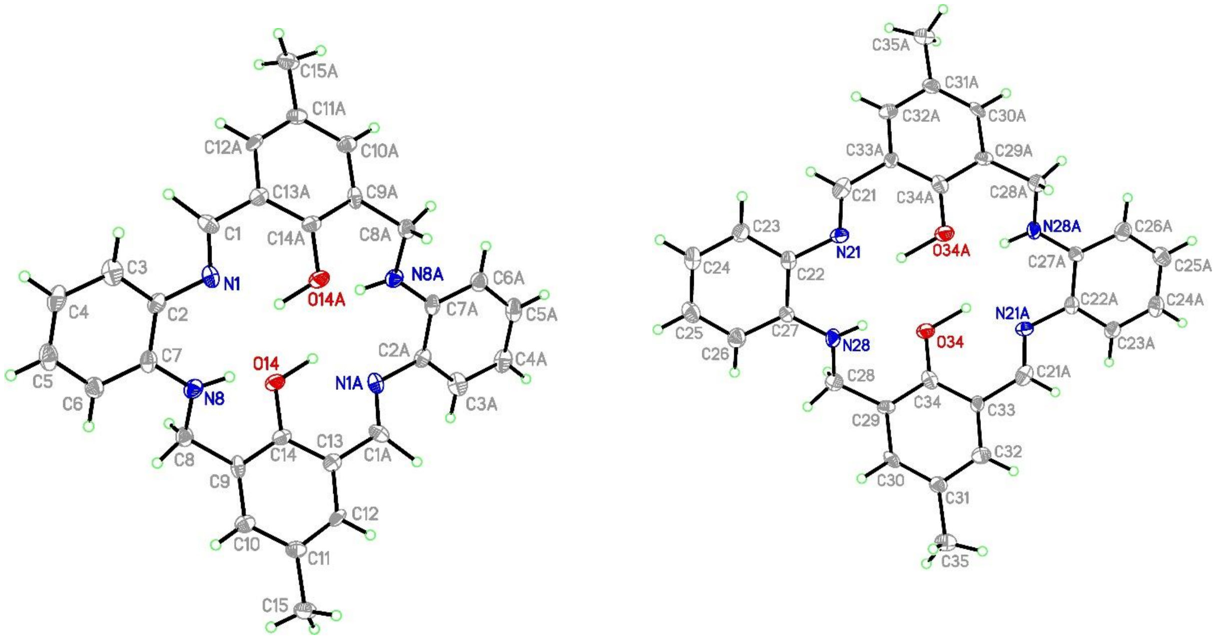

Figure S7. Structure of **L1** with two crystallographically independent molecules resulting from two halves of the macrocyclic compound found in the asymmetric unit. There is given a numbering scheme and thermal ellipsoids at 30% probability

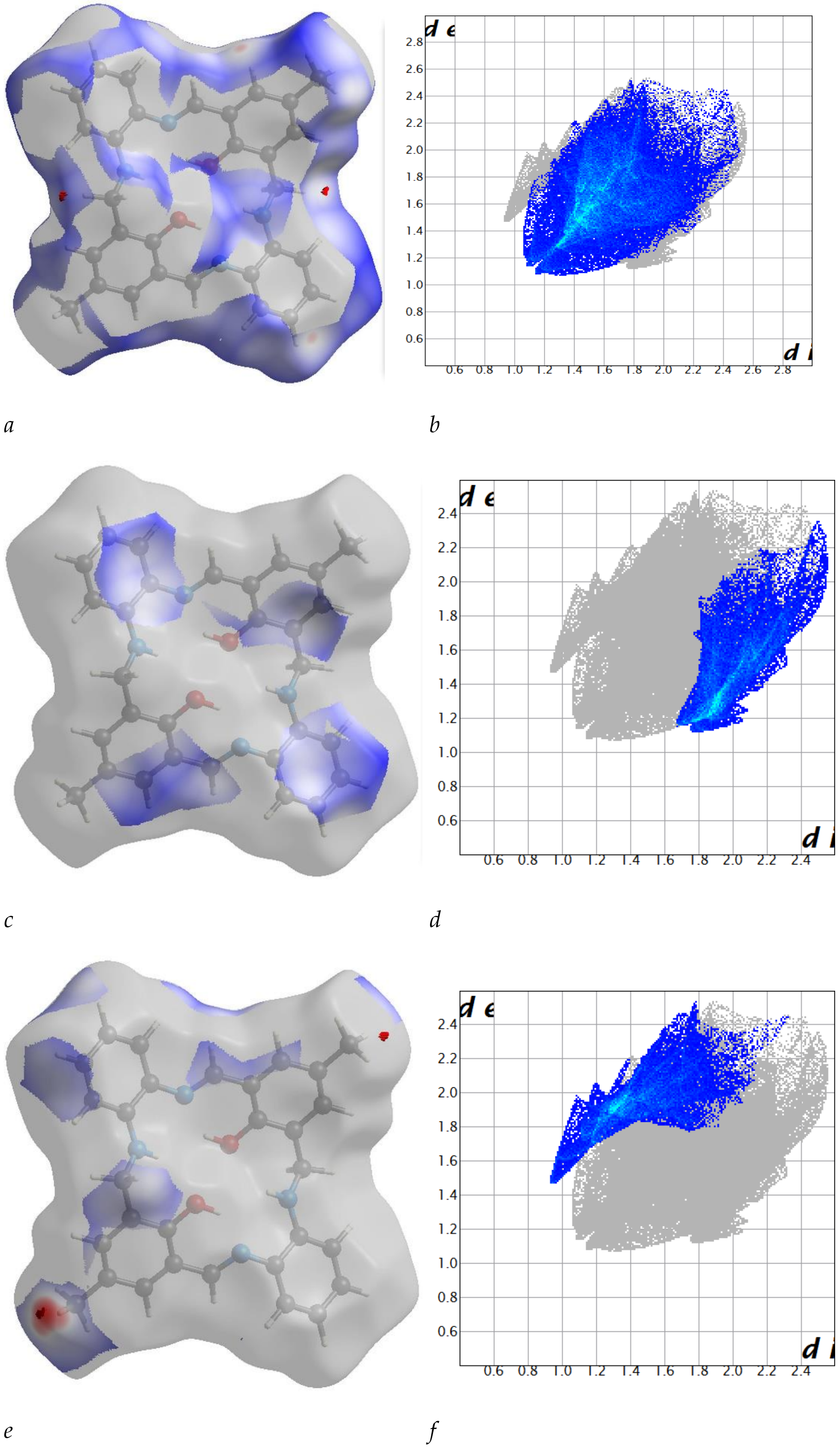

Figure S8. Hirshfeld surfaces and fingerprints of selected interactions created in the crystal network of **L1**: a. Hirshfeld surface for H…H (red markers correspond to the shortest H…H

interactions at 1.15, 1.1), b. fingerprint for H…H (51.1%), c. Hirshfeld surface for C…H, d. fingerprint for C…H (19.3%), e. Hirshfeld surface for H…C (red markers correspond mainly to the spike at 0.95; 1.5), f. fingerprint for H…C (15.2%) for O34 molecule. In brackets, there is given surface area included as a percentage of the total surface area.

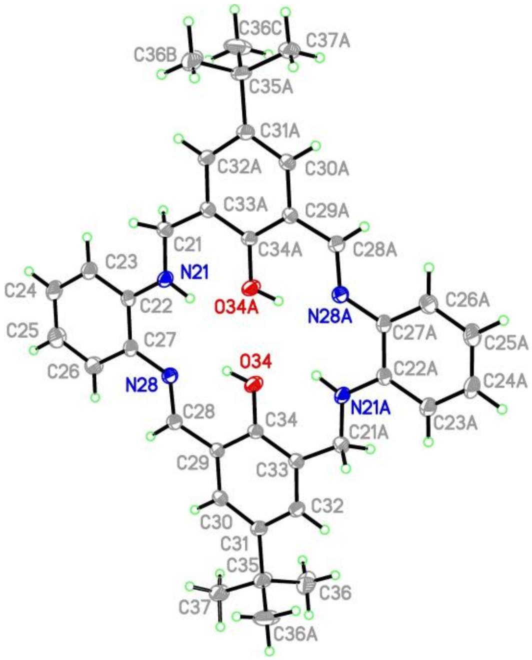

Figure S9. Structure of **L2** with a numbering scheme and thermal ellipsoids at 30% probability.

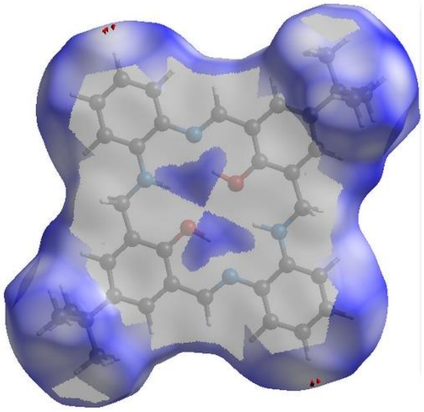

a

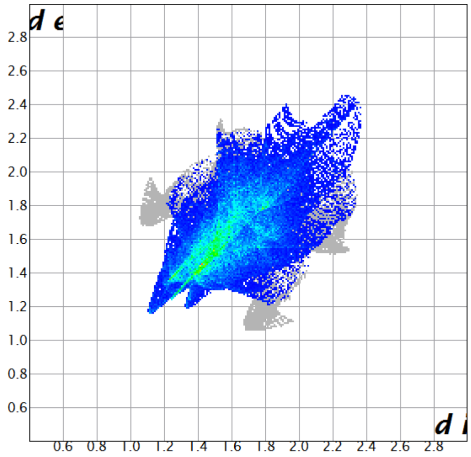

b

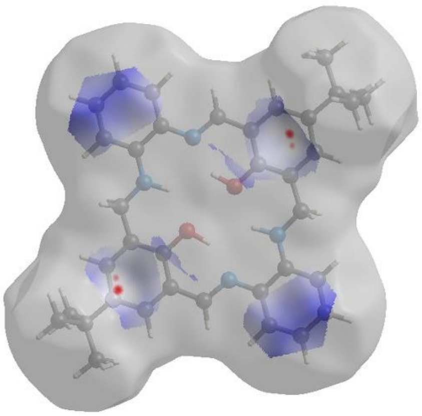

c

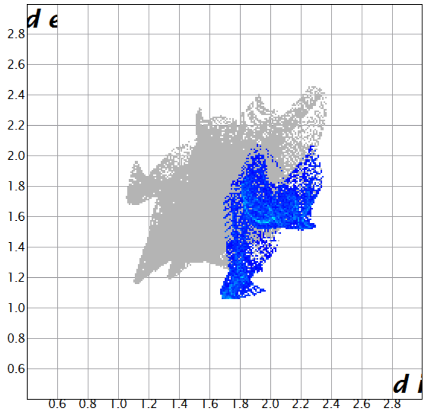

d

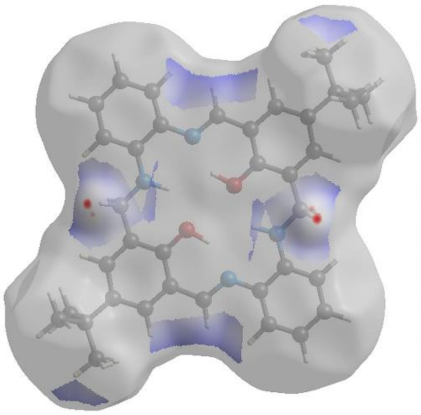

e

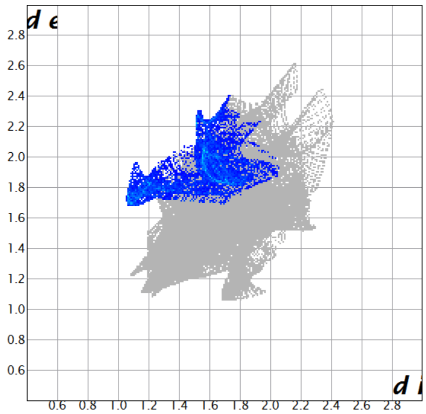

f

Figure S10. Hirshfeld surfaces and fingerprints of selected interactions created in the crystal network of **L2**: a. Hirshfeld surface for H…H (red markers correspond to the large spike at 1.1; 1.15), b. fingerprint for H…H (65.4%), c. Hirshfeld surface for C…H, d. fingerprint for C…H (12.4%), e. Hirshfeld surface for H…C, f. fingerprint for H…C (10.5%) for O34 molecule. In brackets, there is given surface area included as a percentage of the total surface area.

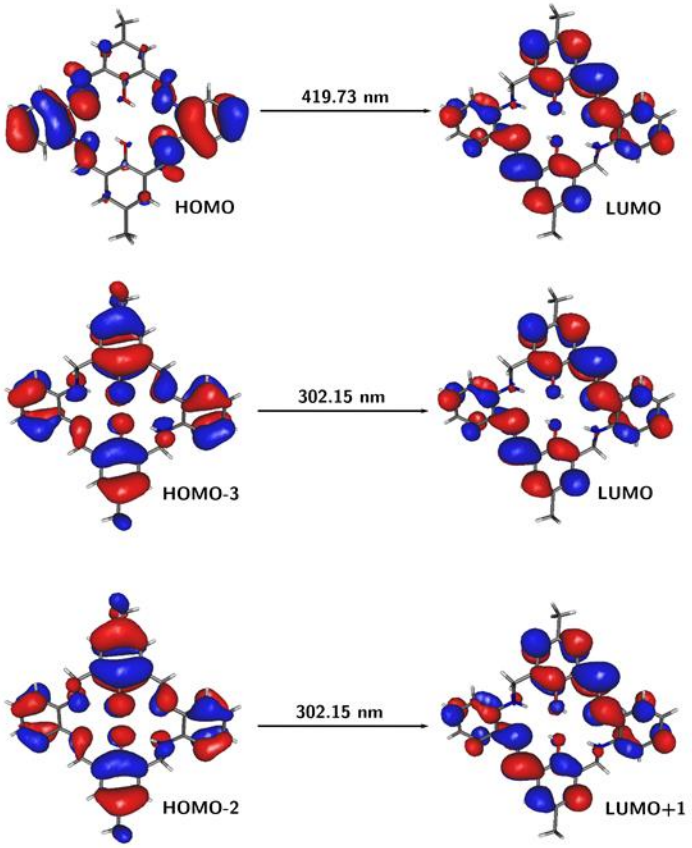

Figure S11. Frontier molecular orbitals of **L1** for the most intensive transitions (PBE0/6-311++G(d,p)/PCM(acetonitrile)).

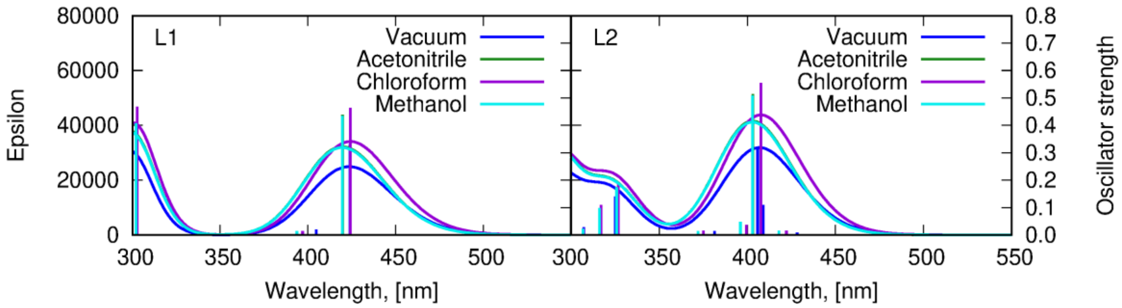

Figure S12. Absorption spectrum of **L1**, **L2** calculated with PBE0/6-311++G(d,p) approach in vacuum and in solvents

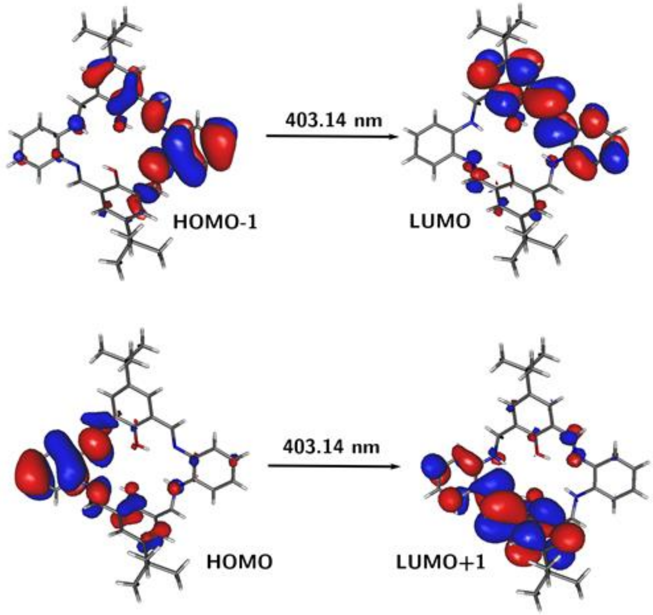

Figure S13. Frontier molecular orbitals of **L2** for the most intensive transitions (PBE0/6-311++G(d,p)/PCM(acetonitrile)).

Table S1. Crystal data and structure refinement for **L1** and **L2**.

Identification code	**L1**	**L2**
Empirical formula	$C_{30} H_{28} N_4 O_2$	$C_{36} H_{40} N_4 O_2$
Formula weight	476.56	560.72
Temperature [K]	293(2)	100(2)
Wavelength [Å]	0.71073	1.54184
Crystal system, space group	Monoclinic, $P2_1/c$	Orthorhombic, Pbam

Unit cell dimensions [Å] and [°]	a = 11.911(2) α= 90<br>b = 8.9920(15) β= 93.153(19)<br>c = 22.463(5) γ = 90	a = 28.2607(7) α= 90<br>b = 15.2760(3) β= 90<br>c = 6.7643(2) γ = 90
Volume [$Å^3$]	2402.3(8)	2920.22(13)
Z, Calculated density [$Mg{\cdot}m^{-3}$]	4, 1.318 Mg/m^3	4, 1.275
Absorption coefficient [mm^{-1}]	0.084	0.625
F(000)	1008	1200
Crystal size [mm]	0.630 x 0.210 x 0.080	0.160 x 0.070 x 0.040
Theta range for data collection [°]	2.427 to 26.364	3.289 to 74.463
Limiting indices	-14<=h<=14<br>-11<=k<=11<br>-28<=l<=28	-35<=h<=34<br>-19<=k<=19<br>-8<=l<=7
Reflections collected/unique	5698 / 5698 [R(int) = 0.1042]	16898 / 3256 [R(int) = 0.0416]
Completeness to theta = 29.732° [%]	99.9	100.0
Max. and min. transmission	0.993 and 0.949	0.975 and 0.907
Refinement method	Full-matrix least-squares on F^2	Full-matrix least-squares on F^2
Data/restraints/parameters	5698 / 2 / 335	3256 / 2 / 251
Goodness-of-fit on F^2	0.887	0.987
Final R Indices [I>2sigma(I)]	R_1 [a] = 0.0746, wR_2 [b] = 0.1856	R_1 [a] = 0.0538, wR_2 [b] = 0.1507

R indices (all data)	R_1 [a] = 0.1788, wR_2 [b] = 0.2247	R_1 [a] = 0.0657, wR_2 [b] = 0.1622
Largest diff. peak and hole [eÅ^{-3}]	0.318 and -0.241	0.366 and -0.361

[a] $R1 = \Sigma||F_0| - |F_c||/\Sigma|F_0|$

[b] $wR2 = [\Sigma w(F_0^2 - F_c^2)^2/\Sigma(w(F_0^2)^2)]^{1/2}$

Table S2. Selected bond length [Å] and valence angles [°] for the **L1**.

	**L1**		
C1-N1	1.282(7)	N8-C8	1.455(7)
N1-C2	1.436(8)	C7-N8	1.362(8)
C1-C13^{i}	1.460(9)	C8-C9	1.501(9)
C21-N21	1.295(7)	N28-C28	1.442(7)
N21-C22	1.416(7)	C27-N28	1.371(7)
C21-C33^{ii}	1.464(9)	C28-C29	1.508(9)
	Valence angles [°]		
C1-N1-C2	118.4(6)	C21-N21-C22	119.3(6)
N1-C1-C13^{i}	124.2(7)	N21-C21-C33^{ii}	124.1(7)

(**L1**) ^{i} -x+1,-y+1,-z ^{ii} -x+2,-y+2,-z

Table S3. Relevant photophysical data of studied compounds, (λ_{em}, λ_{ex} nm, λ[nm] (ε [dm^3 mol^{-1} cm^{-1}]). Bp=8

**Compound**	**Solvent**	**λ_{ex} [nm]**	**λ_{em} [nm]**	**Fluorescence Intensity a. u.**	**λ[nm] (ε [dm^3 mol^{-1} cm^{-1}])**	**A**	**C**
**L1**	acetonitrile	295<br>350	454<br>459	1491630<br>1349115	338(16176)<br>346(16294)<br>382(16353)	0,275<br>0,277<br>0,278	1,7*10^{-5}
	chloroform	295<br>350	498<br>500	1939430<br>2957245	338(16765)R<br>350(17117)<br>382(15823)	0,285<br>0,291<br>0,269	1,7*10^{-5}

	methanol	295<br>350	497<br>498	2792725<br>4940850	342(16800)	0,336	$2*10^{-5}$
	benzene	295<br>350	516<br>514	968195<br>2100280	336(14529)<br>350(14765)<br>390(14412)	0,247<br>0,251<br>0,245	$1,7*10^{-5}$
**L2**	acetonitrile	295<br>350	452<br>453	3270265<br>2621965	340(19529)<br>382(20882)	0,332<br>0,355	$1,7*10^{-5}$
	chloroform	295<br>350	492<br>494	5620800<br>6858510	338(27882)<br>346(28471)<br>380(27176)	0,474<br>0,484<br>0,462	$1,7*10^{-5}$
	methanol	295<br>350	493<br>495	2646015<br>3994280	342(22214)	0,311	$1,4*10^{-5}$
	benzene	295<br>350	517<br>514	3085945<br>5024360	342(16941)<br>390(16176)	0,288<br>0,275	$1,7*10^{-5}$

Table S4. Theoretical PBE0/6-311++G(d,p)/PCM(ACN) vertical excitation wavelengths λ [nm] for most intensive transitions together with the corresponding oscillator strengths f and the orbital contributions for investigated species.

**L1**		
λ [nm]	f	Orbitals (contribution)
419.73	0.4374	HOMO->LUMO (63%)
302.15	0.4114	HOMO-3->LUMO (47%)<br>HOMO-2->LUMO+1 (40%)
**L2**		
403.14	0.5140	HOMO-1->LUMO (63%)
325.87	0.1753	HOMO-2->LUMO (67%)

Table S5. Relevant fluorescent data of studied compounds in the solid state (λ_{em}, λ_{ex}) [nm].

**Compound**	**λ_{ex} [nm]**	**λ_{em} [nm]**
**L1**	295	548
	350	552
**L2**	295	549
	350	561

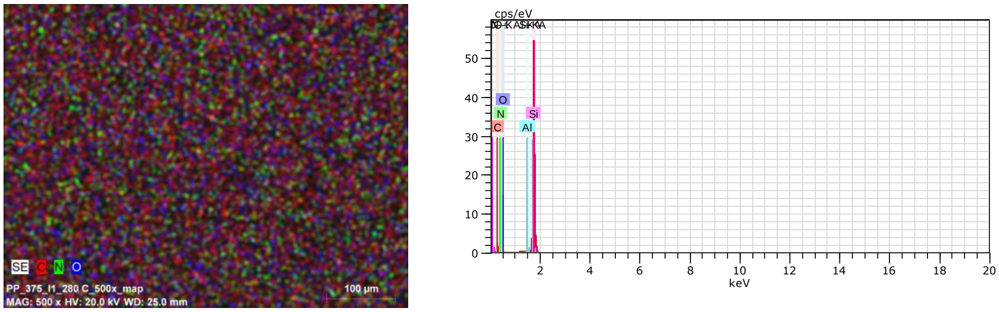

Figure S14. SEM images of **L1**/Si maping scanning size 100 µm.

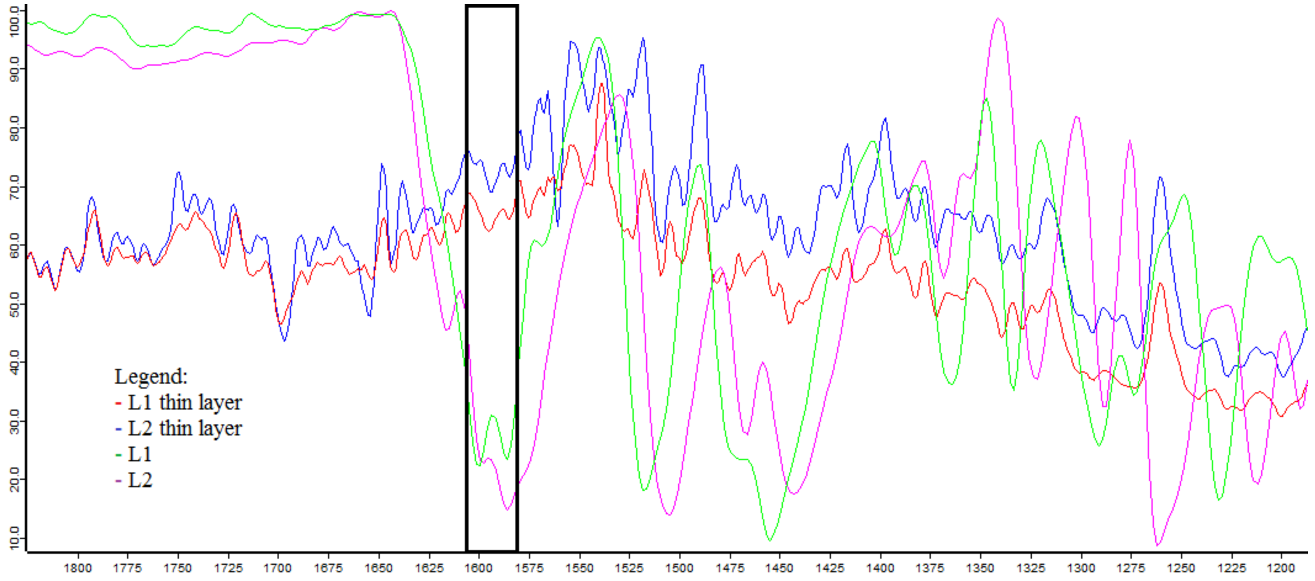

Legend:
- L1 thin layer
- L2 thin layer
- L1
- L2

a)

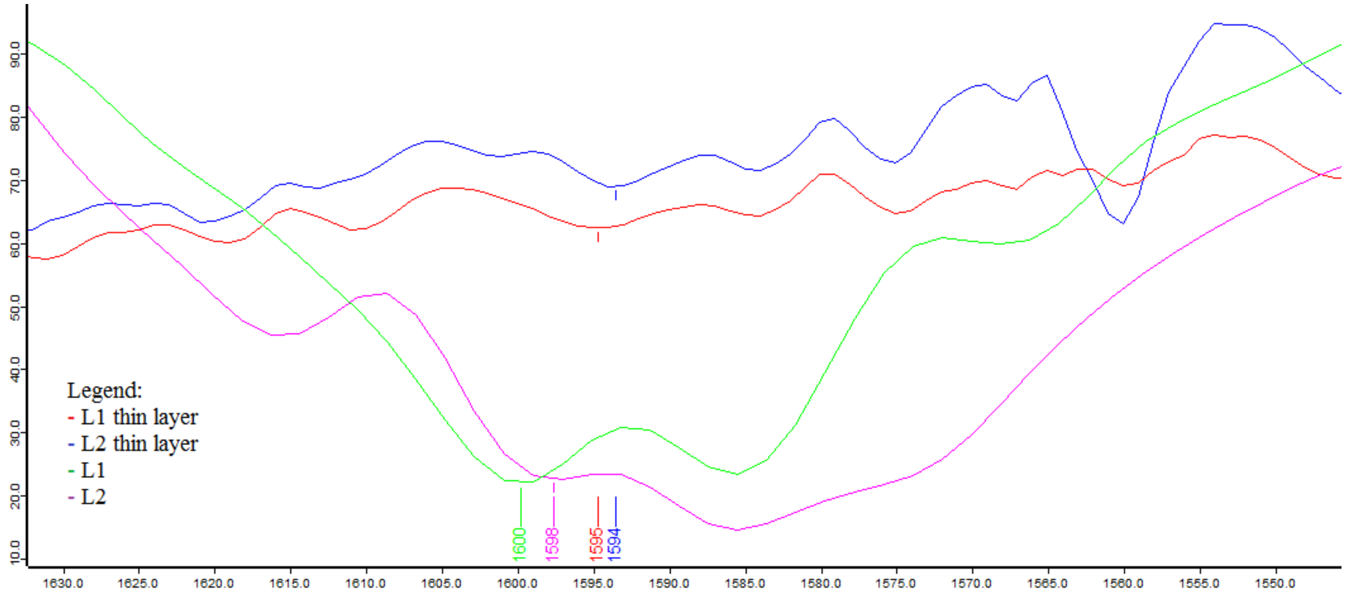

Legend:
- L1 thin layer
- L2 thin layer
- L1
- L2
1600
1598
1595
1594

b)

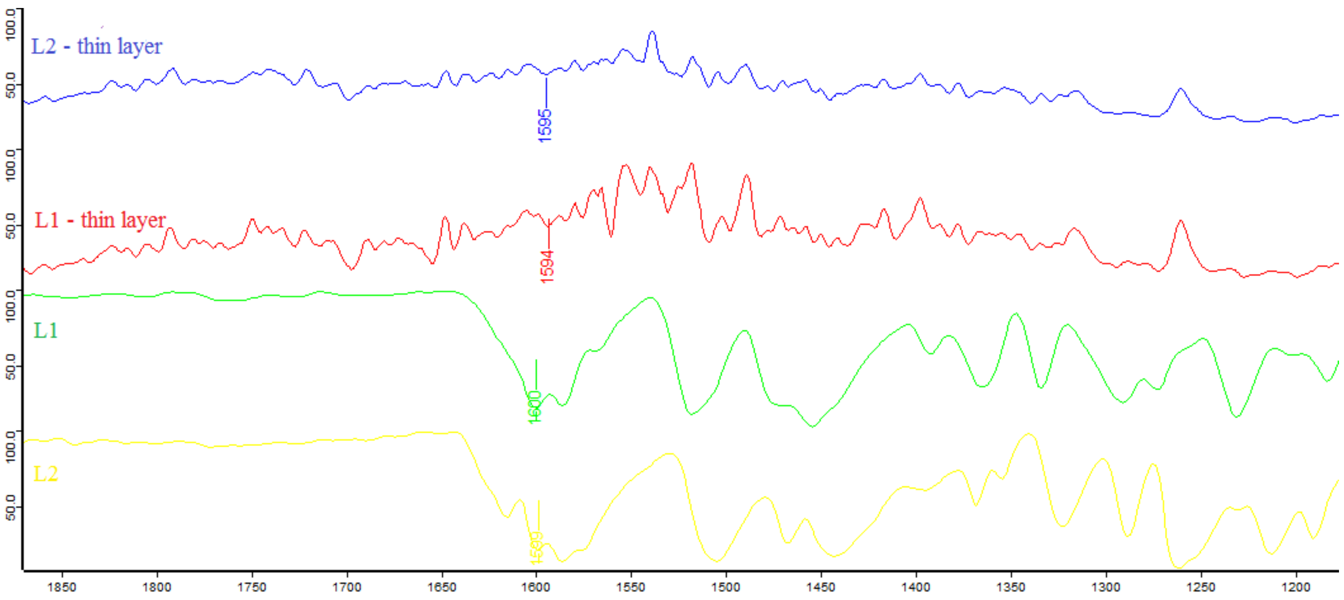

c)

Figure S15. (a), (b) and (c) DRIFT spectra of **L1** and **L2** samples and **L1**/Si and **L2**/Si.
